# Conditional clustering of temporal expression profiles

**DOI:** 10.1186/1471-2105-9-147

**Published:** 2008-03-11

**Authors:** Ling Wang, Monty Montano, Matt Rarick, Paola Sebastiani

**Affiliations:** 1 Novartis Vaccines and Diagnostics, Emeryville, CA 94608, USA; 2 Section of Infectious Diseases, Center for HIV-1/AIDS Care and Research, Boston University School of Public Health, Boston, MA 02118, USA; 3 Department of Biostatistics, Boston University School of Public Health, Boston, MA 02118, USA

## Abstract

**Background:**

Many microarray experiments produce temporal profiles in different biological conditions but common cluster techniques are not able to analyze the data conditional on the biological conditions.

**Results:**

This article presents a novel technique to cluster data from time course microarray experiments performed across several experimental conditions. Our algorithm uses polynomial models to describe the gene expression patterns over time, a full Bayesian approach with proper conjugate priors to make the algorithm invariant to linear transformations, and an iterative procedure to identify genes that have a common temporal expression profile across two or more experimental conditions, and genes that have a unique temporal profile in a specific condition.

**Conclusion:**

We use simulated data to evaluate the effectiveness of this new algorithm in finding the correct number of clusters and in identifying genes with common and unique profiles. We also use the algorithm to characterize the response of human T cells to stimulations of antigen-receptor signaling gene expression temporal profiles measured in six different biological conditions and we identify common and unique genes. These studies suggest that the methodology proposed here is useful in identifying and distinguishing uniquely stimulated genes from commonly stimulated genes in response to variable stimuli. Software for using this clustering method is available from the project home page.

## Background

Cluster analysis of gene expression data is commonly used to group gene expression measurements, cross-sectionally or longitudinally, into categories of genes that have similar patterns of expression. Various clustering methods have been proposed to analyze microarray data [1-4], and hierarchical and k-means clustering have been applied to the discovery and characterization of the regulatory mechanisms of several processes and organisms [5-10]. Time course microarray experiments allow investigators to look at the behaviors of genes over time and hence introduce another dimension of observation [11]. In the past few years, several methods for clustering temporal gene expression data have been proposed that use autoregressive models (CAGED) [12], hidden Markov models [13], mixture models with variations of the EM algorithm [14] or B splines [15], and Bayesian hierarchical models with an agglomerative search using smoothing splines [16]. However, it has been suggested that autoregressive models [12] and some other clustering procedures [13,17] are better suited to cluster long temporal gene expression data, possibly measured at regularly spaced time points [18]. We introduced an extension to CAGED that overcomes this limitation and is more suitable to cluster short gene expression profiles in [19]. This method uses polynomial models to describe the expression profiles and uses proper prior distributions for the model parameters to make the result of clustering invariant under linear transformations of time [20].

Furthermore, the algorithm finds the optimal number of clusters during the clustering process using a Bayesian decision-theoretic approach.

The common objective of all these different methods is to cluster gene expression temporal profiles observed in one specific biological condition, but in many experiments the temporal expression profiles are observed under different biological conditions: for example Diehn and coauthors [21] measured the gene expression profile of T cells under various activating stimulations to identify those genes that react uniquely to specific activating stimulations. To account for different biological conditions, Storey et al. [22] introduced an F statistic for differential analysis of time course expression data that produced a principled way to find the genes that are expressed differently in two experimental conditions. Here, the objective is different: we are not only interested in discovering the genes that have different dynamics in different experimental conditions. We wish to be able to simultaneously discover these genes and also group them together with other genes that have the same temporal expression profiles.

Other authors have already proposed solutions to this problem. Heard et al. [23] have proposed to extend their model-based clustering of temporal expression profiles in multiple conditions by modeling the concatenated time series. This approach has the advantage of increasing the robustness of clustering by simultaneously using the information from the different experiments. However, this gain of robustness comes at the price that only concatenated time series are clustered, so those genes that have a similar profile in one experimental condition but different profiles in the other conditions will be lost because they are allocated to different clusters. Ng et al [24] proposed a mixture model with random effect that uses the EM algorithm to cluster gene expression data from either time course experiment or experiments with replicates. The flexible nature of linear mixed model gives a unified approach to cluster data collected from various designed experiments and, in principle, this method is applicable to clustering temporal data measured in different conditions. However, similarly to the method in [23], it only clusters the concatenated time series from multiple conditions.

Here we propose an extension of our polynomial-based method to cluster short expression profiles measured in different conditions. This method that we call *conditional clustering *stratifies the data according to the experimental conditions and performs separate cluster analysis within the strata, then attempts to merge the resulting clusters if the merging could improve a Bayesian metrics. Because clustering within each stratum does not use all the information for those genes that have common patterns across more than one experimental condition, we further use an iterative procedure to find those genes that have unique expression profile under a specific condition and genes that have common expression profiles under two, or even more conditions. An application of this novel procedure to real data produces biologically meaningful gene sets.

## Results and discussion

**Figure 1:**
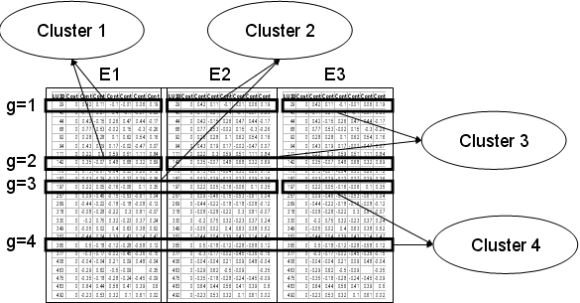
**Example of our approach to cluster temporal expression profiles measured in 3 biological conditions E1, E2 and E3.** Cluster 1 comprises the expression profiles of genes 1 and 2 under the experimental condition E1. Cluster 2 comprises the expression profiles of gene 3 in both experimental conditions E1 and E2, and the expression profile of gene 1 in the experimental condition E2. Cluster 3 comprises the expression profile of gene 1 in the experimental condition E3, and the expression profiles of gene 2 in both experimental conditions E2 and E3. Cluster 4 comprises the expression profiles of gene 4 in all three experimental conditions E1, E2 and E3. The first gene has a unique expression profile in each experimental condition, so this gene reacts specifically (uniquely). Gene 2 has a unique profile in the third experimental condition and common profiles in both experimental conditions E1 and E2. Gene 4 has common profiles in all experimental conditions.

Suppose we have gene expression temporal profiles measured across different conditions. Our objective is to identify those genes that have common profiles in two or more experimental conditions, as well as those genes that have a unique profile in one specific experimental condition. Therefore, our clusters can group the profiles of *different genes *that have similar expression patterns in one or more experimental conditions, but also the profiles of the *same gene *with similar expression patterns in two or more conditions. Figure 1 shows an example. We use a two-step process. First we use the algorithm we introduced in [19] to cluster data in each experimental condition separately. Then we check if any of the resulting clusters from the first step can be merged according to our merging criterion, and introduce an iterative procedure that searches for genes with common patterns of expression across two or more conditions as well as genes with a unique pattern of expression in some particular condition.

### Cluster analysis within conditions

Within each condition, we use the model-based clustering procedure that we introduced in [19]. The procedure assumes that the expression data of *m *genes measured at *n *time points *t*_*i *_are generated from an unknown number of processes and the goal is to group them into a number of clusters so that each cluster contains those genes with expression profiles generated from the same process. Each process is described by a Bayesian model of the log-transformed expression profile $x_{g}=\{x_{gt_{1}},x_{gt_{2}},...,x_{gt_{n}}\}$, where the expected pattern is the polynomial model:

$$x_{gt_{i}}=\mu_{g}+\beta_{g1}t_{i}+...+\beta_{gp}t_{i}^{p}.$$

In matrix notation we can write

(1) *x*_*g *_= *Fβ*_*g *_+ *ε*_*g*_

where $x_{g}={\left(x_{gt_{1}},x_{gt_{2}},...,x_{gt_{n}}\right)}^{T}$, *F *is the *n *× (*p *+ 1) design matrix in which the *i*^*th *^row is $\left(1,t_{i},t_{i}^{2}...,t_{i}^{p}\right)$, *β*_*g *_= (*μ*_*g*_, *β*_*g*1_, ..., *β*_*gp*_)^*T *^is the vector of regression coefficients, and $\varepsilon_{g}={\left(\varepsilon_{gt_{1}},\varepsilon_{gt_{2}},...,\varepsilon_{gt_{n}}\right)}^{T}$ is the vector of errors. We assume that the errors are normally distributed, with $E(\varepsilon_{gt_{i}})=0$ and $Var(\varepsilon_{gt_{i}})=1/\tau_{g}$, for any *t*_*i*_, and *p *is the polynomial order. To complete the specification of the Bayesian model, we assume the standard conjugate normal-gamma prior density for the parameters *β*_*g *_and *τ*_*g *_so that the marginal distribution of *τ*_*g *_and the distribution of *β*_*g*_, conditional on *τ*_*g*_, are

*τ*_*g *_~ Gamma(*α*_1_, *α*_2_)

*β*_*g*_|*τ*_*g *_~ *N*(*β*_0_, (*τ*_*g*_*R*_0_)^-1^)

where *E*(*τ*_*g*_) = *α*_1_*α*_2_, *E*(1/*τ*_*g*_) = 1/((*α*_1 _-1)*α*_2_), and *E*(*β*_*g*_) = *β*_0 _= (*β*_00_, *β*_01_, ..., *β*_0*p*_)^*T*^, and *R*_0 _is the (*p *+ 1) × (*p *+ 1) identity matrix. The prior hyper-parameters *α*_1_, *α*_2_, *β*_0 _are identical for all genes. We use the data of the non-expressed genes to specify the prior hyper-parameters, as suggested in [19,25]. To compare different partitions of the genes, we compute the posterior probability of different clustering models so that, given the observed gene expression profiles, the best clustering model is the one with maximum posterior probability. This method was originally suggested in [12] and works as follows. Let *M*_*c *_denote the model with *c *clusters of gene expression data, where each cluster *C*_*k *_groups the set of expression profiles generated by the same polynomial models with coefficients *β*_*k *_and variance 1/*τ*_*k*_, *k *= 1, ..., *c*. Each cluster contains *m*_*k *_genes that are jointly modeled as

(2) *x*_*k *_= *F*_*k*_*β*_*k *_+ *ε*_*k*_

where the vector *x*_*k *_and the matrix *F*_*k *_are defined by stacking the vectors $x_{k1},x_{k2},...,x_{km_{k}}$ and the regression matrix $F_{k1},F_{k2},...,F_{km_{k}}$ in the following way

(3) $$x_{k}=\left(\begin{array}{c} x_{k1}\\ x_{k2}\\ \vdots \\ x_{km_{k}} \end{array}\right)F_{k}=\left(\begin{array}{c} F_{k1}\\ F_{k2}\\ \vdots \\ F_{km_{k}} \end{array}\right)$$

Note that we now label the vectors *x*_*g *_assigned to the same cluster *C*_*k *_with the double script *k*_*g*_, in which *k *denotes the cluster membership, and *g *= 1, ..., *m*_*k *_is the index for the genes in this cluster. Here *m *= ∑_*k *_*m*_*k*_, *ε*_*k *_is the vector of uncorrelated errors with *E*(*ε*_*k*_) = 0 and *V*(*ε*_*k*_) = 1/*τ*_*k*_.

The posterior probability of the model *M*_*c *_is *P*(*M*_*c*_|*x*) ∝ *P*(*M*_*c*_)*f*(*x*|*M*_*c*_), where *P*(*M*_*c*_) is the prior probability, *x *consists of all the time series data, and *f*(*x*|*M*_*c*_) = ∫ *f*(*x*|*θ*)*f*(*θ*|*M*_*c*_)*dθ *is the marginal likelihood. The vector of parameters *θ *contains all the parameters specifying the model *M*_*c*_, the function *f*(*θ*|*M*_*c*_) is its prior density, and the function *f*(*x*|*θ*) is the likelihood function. Since we assume that the profiles assigned to different clusters are independent, the overall likelihood function is

$$f(x|\theta )=\prod_{k=1}^{c}p_{k}^{m_{k}}f(x_{k}|\beta_{k},\tau_{k})$$

where *p*_*k *_is the marginal probability that a gene expression profile is assigned to the cluster *C*_*k*_. We assume a symmetric Dirichlet distribution for the parameters *p*_*k*_, with hyper-parameters *ηk *∝ *p*_*k*_. and overall precision *η *= ∑_*k*_*η*_*k*_. Then the marginal likelihood *f*(*x*|*M*_*c*_) can be calculated in closed form and is given by the formula

(4) $$\begin{array}{l} f(x|M_{c})=\frac{\Gamma (\eta )}{\Gamma (\eta +m)}\\ \prod_{k=1}^{c}\frac{\Gamma (\eta_{k}+m_{k})}{\Gamma (\eta_{k})}\frac{1}{{\left(2\pi \right)}^{(m_{k}n)/2}}\frac{{\left(\det R_{0}\right)}^{1/2}}{{\left(\det R_{kn}\right)}^{1/2}}\frac{\Gamma (\alpha_{k1n})}{\Gamma (\alpha_{1})}\frac{\alpha_{k2n}^{\alpha_{k1n}}}{\alpha_{2}^{\alpha 1}} \end{array}$$

in which

$$\begin{array}{lll} \alpha_{k1n} & = & \alpha_{1}+\frac{m_{k}n}{2}\\ \frac{1}{\alpha_{k2n}} & = & \frac{-\beta_{kn}^{T}R_{kn}\beta_{kn}+x_{k}^{T}x_{k}+\beta_{0}^{T}R_{0}\beta_{0}}{2}+\frac{1}{\alpha_{2}}\\ R_{kn} & = & R_{0}+F_{k}^{T}F_{k}\\ \beta_{kn} & = & R_{kn}^{-1}(R_{0}\beta_{0}+F_{k}^{T}x_{k}) \end{array}$$

When all clustering models are a priori equally likely, the posterior probability *p*(*M*_*c*_|*x*) is proportional to the marginal likelihood *f*(*x*|*M*_*c*_) that becomes our probabilistic scoring metric.

To make the computation feasible, the same agglomerative, finite-horizon heuristic search strategy introduced by Ramoni et al in [12] is used. This heuristic search orders the merging of profiles by their distance, so that the closest profiles are tested for merging first. The procedure first calculates the *m*(*m *- 1) pair-wise distances for the *m *genes and then attempts to merge the two closest genes into one cluster. If this merging increases the likelihood it is accepted, the two genes are assigned to the same clusters and their profile is replaced by the *average profile *that is a point-by-point average of the expression of the two genes. The same procedure is repeated for the new set of *m *- 1 profiles. If this merging is not accepted, the heuristic tries to merge the next second closest genes to see if their merging increases the likelihood or not. This procedure continues until an acceptable merging is found, otherwise it stops. This strategy automatically determines the best number of clusters when it does not find a pair of profiles to be merged into the same cluster and therefore stops. Note that the decision to merge profiles is based on the posterior probability and the distance between profiles is only used to speed up computations. In the implementation we have two possible choices of distances: the Euclidean distance and the negative of the correlation coefficient.

To find the best polynomial order, we repeat the conditional cluster analysis for different values *p*, and compute the Bayesian Information Criterion (BIC) [20] of the cluster set for each *p *and the value that optimizes the BIC is chosen. Let *q *be the number of parameters in the model, then *q *= *c*(*p *+ 2) when the model does not have intercept and *q *= *c*(*p *+ 3) when the model has intercept. The BIC of the clustering model *M*_*c *_is

(5) *BIC *= -2 log *f*(*x*|*M*_*c*_) + *q *log(*n*)

where *f*(*x*|*M*_*c*_) is the marginal likelihood of the model specified in Equation 4. We use the same *p *for clustering in each condition and the final merging of all conditions to ensure consistency of the overall clustering model.

We evaluated this clustering algorithm in three simulated data, and we demonstrated that this algorithm performs well in identifying the correct number of clusters as well as the correct cluster assignments [19]. These results are consistent with other evaluations, where we showed that the combination of a distance driven search with a Bayesian scoring metrics leads to correctly identifying clusters in an efficient way [26,27]. The results were compared to a competing program STEM [18] which is also designed for clustering short temporal gene expression data.

### Cluster analysis across all experimental conditions

**Figure 2:**
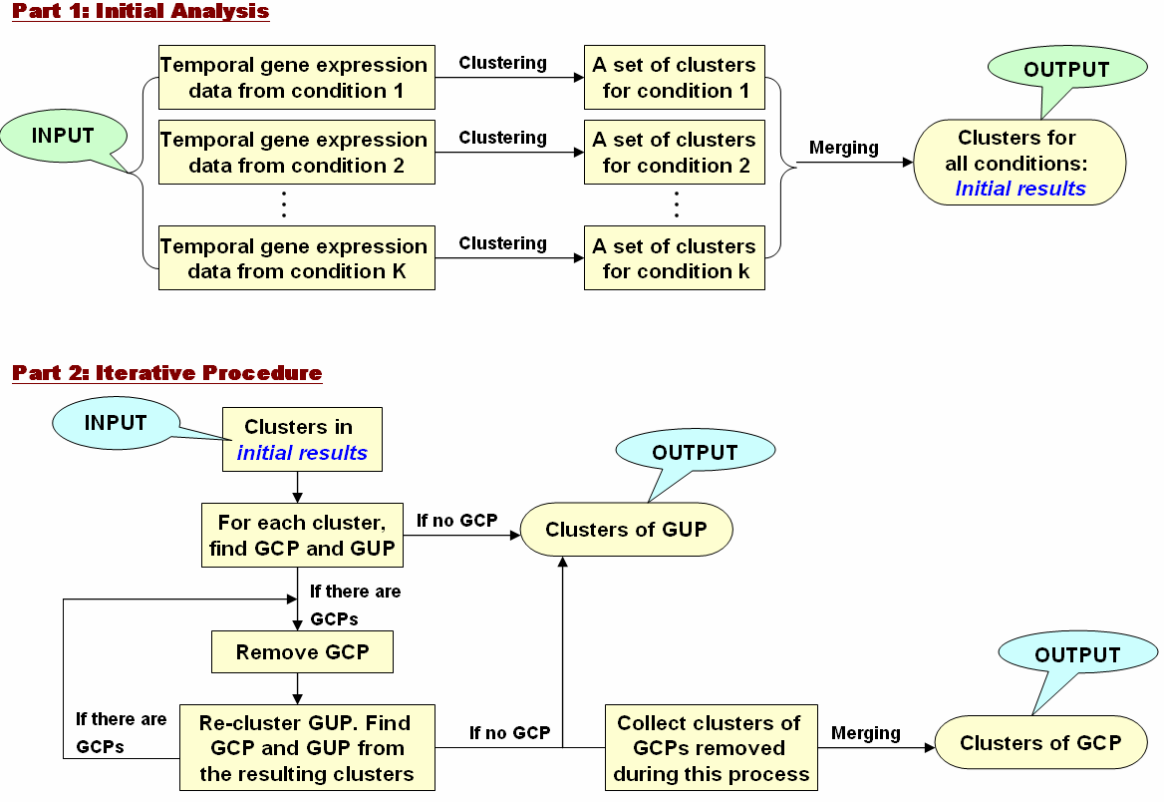
**Flow chart for the two-step procedure for finding GUP and GCP.** Here 'clustering' means performing the cluster analysis method we proposed here, and 'merging' means trying to merge the clusters from the previous step using the cluster analysis method we proposed here.

We consider next the task of clustering temporal gene expression data across several experimental conditions. The cluster analysis within experimental conditions generates a set of clusters and the first step of the conditional clustering method is to merge them thus producing a possibly smaller set of clusters that we term *initial results *(See Figure 2, part 1). We note that the analysis in each biological condition is conducted independently of the data measured in the other conditions, and this is equivalent to assuming that all genes respond "uniquely" to each biological condition. However, some of these initial clusters may contain genes with the same expression profile across different conditions and using this information may produce more robust results. Our conjecture is that we can find *genes *that have a *unique *expression *profile *for a specific biological condition (GUP), and *genes *that have a *common *expression *profile *across two or more experimental conditions (GCP). GCP are "commonly affected" by two or more conditions because they exhibit the same expression profiles that are assigned to the same cluster. On the other hand, GUP are "uniquely affected" by a specific condition and their profile will not be assigned to clusters containing the profiles of the same gene in other conditions. Biologically, GUP are those genes that one would target when looking for an expression profile that characterizes a particular experimental conditions. GCP, on the other hand, would be those genes with robust expression across two or more of the experimental conditions. To identify the GCP and GUP we recursively search for genes whose profile appears only once in a cluster as shown in part 2 of Figure 2. The overall conditional clustering works as follows:

1. Cluster the gene expression profiles within each experimental condition.

2. Use all the clusters generated in step 1 as input of a new cluster analysis across all conditions using the same algorithm. During this step we try the merging of these initial clusters using the marginal likelihood in Equation 4 as scoring metric until no merging can improve the likelihood.

3. Identify the GCP and GUP in the clusters derived in step 2. If there are no GCP, so there are no clusters merging the profiles of the same gene in two or more conditions, go to step 4. If there are GCP go to step 5.

4. There are no GCP. The resulting clusters are the final clusters for GUP.

5. Remove those GCP from the analysis, but keep their cluster assignments. Re-cluster the remaining data containing GUP using our clustering algorithm.

6. Identify the GCP and GUP in the clusters produced at step 5. If there are no GCP, go to step 7. If there are GCP go to step 5.

7. Take the clusters containing the GCP removed during the iterations, try to merge these clusters of GCP to see if the merging could improve the marginal likelihood. The resulting clusters are the final clusters for GCP.

The results from this procedure give us two sets of clusters: clusters of GCP and clusters of GUP. The GCP clusters contain expression profiles for genes that have the same behavior in at least two experimental conditions. The GUP clusters have expression profiles of genes that have unique expression patterns in a particular experimental condition. We should notice that there are multiple conditions here and to choose the optimal polynomial order *p *we proceed as follows. First, for a fixed *p*, we perform the clustering in each condition, then we try to merge the clusters from all the conditions if that improves the likelihood, as mentioned above, and this is the initial results. The optimal *p *is selected by comparing BIC of the various initial results using *p *= 1, ..., *n *- 1.

### Evaluation

We conducted three simulation studies to evaluate the performance of the proposed Bayesian conditional clustering algorithm. The first two simulations examine the effects of sample size and variability on the accuracy of the cluster produced as initial results. The third simulation examines the effectiveness of the whole clustering algorithm in finding GCP and GUP. In all three simulations we generated normalized patterns.

### Simulations 1

#### Data

**Figure 3:**
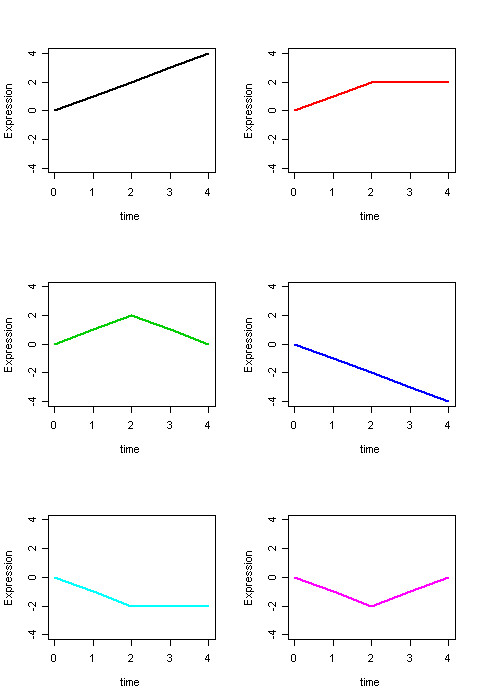
The six baseline patterns used to generate the data in the first two simulation studies.

In the first simulation study, we generated two linear and four non linear patterns plus a flat pattern to represent a total number of seven clusters. Figure 3 shows the first six baseline patterns. We used the seven patterns to generate temporal profiles by adding a normal random noise with mean 0 to the baseline pattern and we varied the variance to assess the effect of variability on the clustering results. As shown in Figure 3, the series were assumed to be observed at five time points: 0, 1, 2, 3 and 4. To examine the effect of different cluster sizes on the precision of the algorithm, we generated different test sets in which the number of temporal profiles per pattern was 350, 700, 1050, 1400, 1750 and 2100, equally distributed across clusters. For each sample size, we also varied the variability using variances that range from 0.2 to 1 by 0.2. Using these different parameter values, we generated 30 sets of temporal expression profiles. We then split the data in each of these 30 sets in two groups as follows. We generated two binary covariates, the first covariate represented the two experimental conditions of a control and a test group, while the second covariate was generated as a Bernoulli variable with probability 0.5. To represent the scenario in which the biological conditions affect the temporal profiles, the series generated using the first three patterns were all assigned to the control group, and the series generated from the other three patterns were assigned to the test group. The seventh 'pattern', or the noise, had half profiles assigned to the control group and half profiles assigned to the test group. In this way there are no GCP in the datasets as no genes have the same pattern in both control and test group except for the background noise. Furthermore, to represent the scenario in which the biological conditions do not affect the expression profiles, the series were partitioned in two groups defined by the randomly generated values of the second covariate.

#### Metrics

We clustered the simulated data using the conditional clustering algorithm and used a statistic proposed by Rand [28] to evaluate the similarity between the results and the true cluster assignment. The rationale of this statistic is that, given two sets of clusters, the pairs of objects that are either assigned to the same cluster or split across clusters in both sets show similar cluster assignments, so the statistic is simply the proportion of pairs of objects that are assigned consistently in the two sets. We borrow the example used in [28] to describe this statistic more in details. Suppose we wish to cluster six objects, say *a, b, c, d, e, f*, and consider two ways of grouping them: group 1 consists of the two clusters (*a, b, c*), (*d, e, f*), and group 2 consists of the three clusters (*a, b*), (*c, d, e*), and (*f*). The six objects can be grouped into 15 possible pairs and we can label the elements of each of the 15 pairs as either "assigned to the same cluster" in both groups, "assigned to different clusters" in both groups, or mixed in the other cases, based on the cluster assignments in the two groups. For example, the pair *ab *is assigned to the same cluster in both groups, the pair *ac *is mixed because it is assigned to the same cluster in the first group but *a *and *c *are assigned to different clusters in the second group, and the pair *ad *is split across two clusters in both groups. In the two groups above the two pairs *ab, de *are assigned to the same clusters, and the seven pairs *ad, ae, af, bd, be, bf, cf *are split across clusters in both groups so that Rand statistic is 9/15 = 0.6.

#### Results

**Figure 4:**
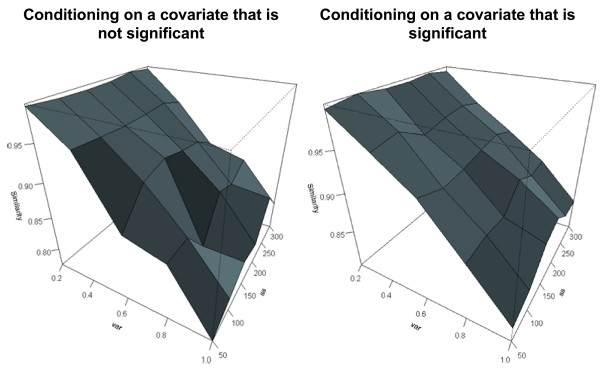
**Similarity of the clusters found by the conditional clustering algorithm compared to the true cluster assignments.** The figure on the left shows Rand statistic as a function of the variance (variable "var") and sample size (variable "ss"), when the clustering was done conditionally on a covariate that is not associated with the temporal patterns. The figure on the right shows Rand statistic when the clustering was conducted conditionally on a covariate that is associated with the temporal patterns. Large values of the statistic show a large agreement with the model used to generate the data.

**Figure 5:**
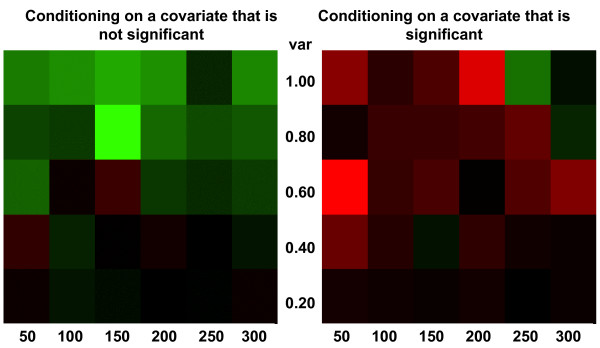
**Heatmaps displaying the difference of Rand statistic between conditional and unconditional clustering in the first set of simulations.** Green cells denote negative values and hence conditional clustering produces clusters with lower accuracy compared to unconditional clustering. Red cells denote positive values and hence conditional clustering produces clusters with higher accuracy compared to unconditional clustering. Black cells show no difference. The intensity of the color shows the magnitude of the number as shown by the legend. The horizontal axis represents the number of simulated patters per cluster, and the vertical axis represents the variance.

The results of the simulation are summarized in Figure 4, which shows that the precision of the conditional clustering algorithm as measured by Rand statistic is decreasing for increasing variance, and increasing for increasing sample size. The cluster precision is however always greater than 80%, thus suggesting that even with patterns that change by more than 2 folds because of noise (variance equal to 1) and relatively small sample sizes (50 patterns per cluster), more than 80% of pairs are assigned to clusters consistently with the data generation model. Note that conditioning on a covariate that is not associated with the expression profiles may slightly decrease the accuracy. To examine the effect of conditioning on the biological condition compared to ignoring this information, we also clustered the time series in all the simulated sets using the unconditional clustering algorithm that ignores covariates [19]. We estimated Rand statistic and then compared the difference in similarity between the conditional and unconditional clustering. We display the results using the heatmaps in Figure 5 that show that conditioning on an irrelevant variable (map on the left) may lead to a loss of accuracy when the variability is large, but conditioning on an informative variable (map on the right) increases the accuracy, particularly when the sample size is small. When the data have smaller variability (variance = 0.2) conditional and unconditional methods have similar performance.

### Simulation 2

**Figure 6:**
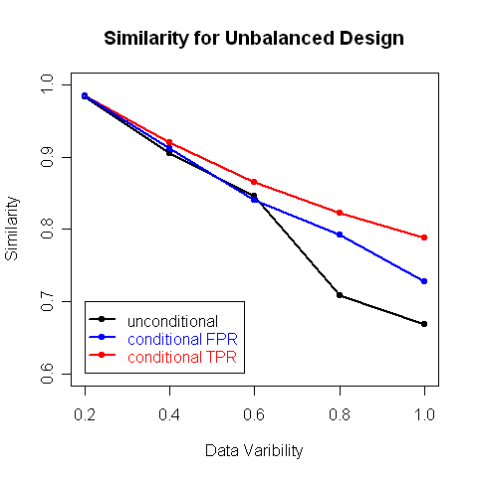
Similarities of the clusters generated by the conditional clustering algorithm with the true cluster assignments in the second set of simulations.

In the second simulation we used the same generating patterns, and same variance structure, but we fixed the total sample size to be 1400 and varied the number of time series generated from each pattern. We combined the data with the two types of covariates as in the first set of simulations, and we clustered the two data sets using the conditional clustering algorithm and the unconditional one. The results are shown in Figure 6, which plots the similarities with the true cluster assignments for the three sets of results. As expected, we can see that conditional clustering (results in red and blue) systematically performs better than unconditional clustering (black line), even when there is no association between the biological conditions and the time series. Furthermore, as the variability increases, the accuracy of clustering decreases.

### Simulation 3

**Figure 7:**
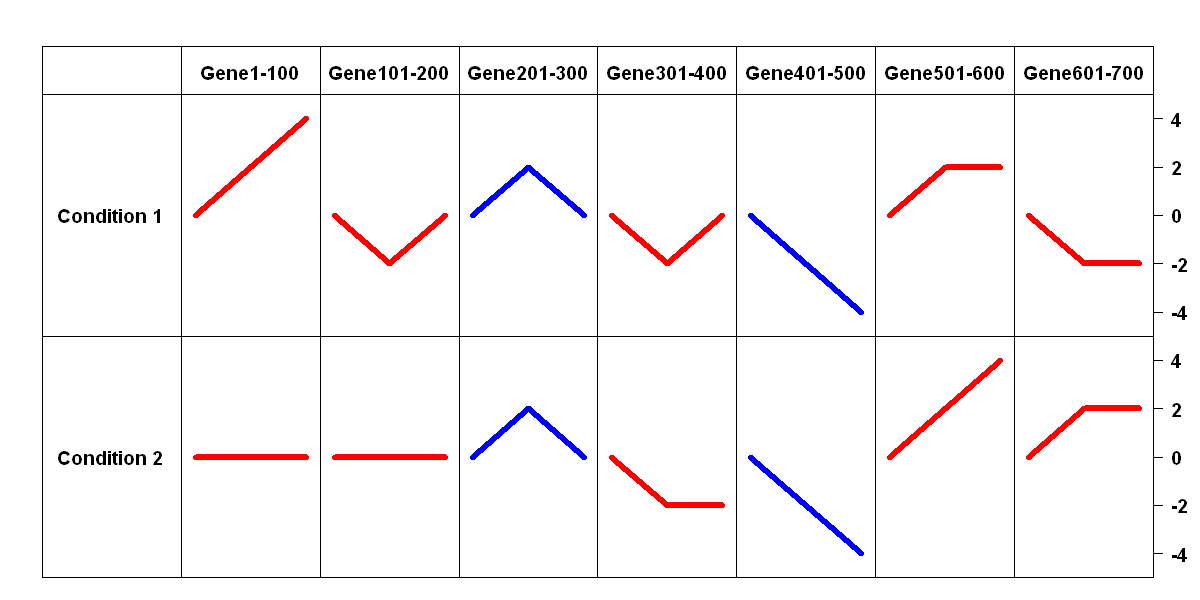
**Patterns used to generate data in the third simulation, and assignment to one of the two conditions.** Red patterns are those of the GUP, and the blue patterns are those of the GCP. Labels on the right of the table show the magnitude of the patterns.

The third simulation study was designed to evaluate the effectiveness of this new conditional clustering algorithm in finding GCP and GUP. We simulated expression data for 700 genes under two experimental conditions. Of these 700 genes, 200 had common expression profiles in these two conditions and 500 had unique expression profiles. Figure 7 shows the true expression patterns of these 700 genes in the two experimental conditions. The true number of clusters was seven in this simulation design, as we can see in Figure 7, and genes 201–300, 401–500 had the same profile in conditions 1 and 2, while the remaining genes had different profiles in the two conditions. The data generation mechanism was the same as the first two simulations.

**Figure 8:**
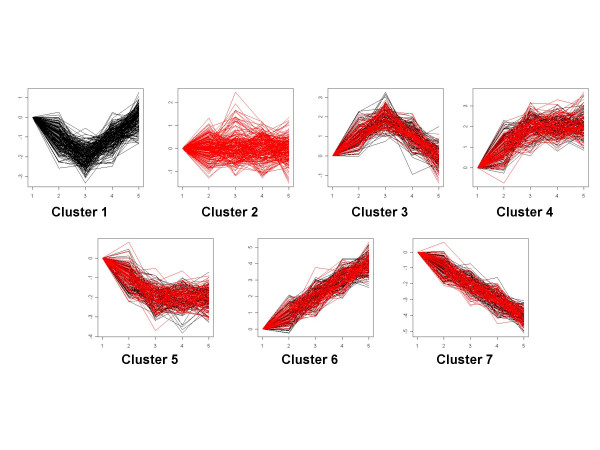
**Simulation 3: The seven clusters resulting from the initial clustering of the simulated data before finding GCP and GUP.** Black series are expressions in condition 1. Red series are expressions in condition 2. Note that we allow the ranges of the y-axis differ for different clusters for better reflection of the cluster shapes.

First, we clustered these simulated profiles to see if our algorithm could successfully find the correct number of clusters. Specifically, we clustered the simulated data under each of the two conditions separately, then we checked if any of the clusters generated conditional on the experimental condition could be merged. From these initial results, we found a total of seven clusters, which are shown in Figure 8. The optimal polynomial order found using the BIC is 4, and the BIC of this optimal model is 14856.16. Of these 1400 expression profiles, 47 profiles were allocated to the wrong cluster, with 13 false negatives (genes with non-flat pattern allocated to flat cluster, cluster 2) and but no false positive (flat pattern genes allocated to clusters with non-flat pattern).

Then we performed the iterative procedure in part 2 of the flow chart in Figure 2 to identify the GCP and GUP in this simulated dataset. The results of this iterative procedure are shown in Table 1. Of these 700 simulated genes, 15 are wrongfully classified as GCP, and 21 are wrongfully classified as GUP. The error rate of the classification is 5%.

**Table 1:** Cross classification table of the iterative clustering results for the simulated 700 genes.

		Our Results Assignment		
		
		GCP	GUP	Total
True Assignment	GCP	179	21	200
	GUP	15	485	500
		
	Total	194	506	700

### Application

We applied our new clustering algorithm to the gene expression data from [21]. The experiment used cDNA microarrays to study the genomic expressions of human T cells in an experimental control condition, and in response to stimulations of CD3, CD28, their co-stimulation CD3/CD28, lectin phytohemagglutinin (PHA), and a combination of the calcium ionophore ionomycin and the phorbol ester phorbol 12-myristate 13-acetate (PMA/lo). In each of these conditions, the expression profile of human T cells was observed at 0, 1, 2, 6, 12, 24, 48 hours. We used the expression profiles analyzed in [21], but removed the profiles with missing data so that we analyzed the expression levels of 2,362 genes.

**Figure 9:**
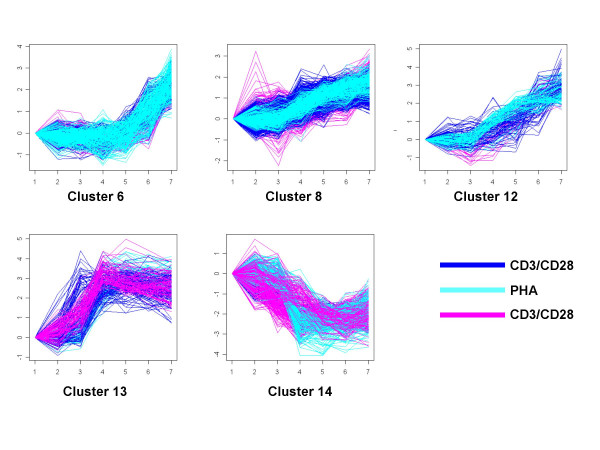
The five clusters related to CD3/CD28, PHA and PMA/lo.

In their original analysis, Diehn and coauthors analyzed the concatenated expression profiles, and concluded that CD3, CD3/CD28, PHA andPMA/lo determined essentially the same response, and only CD28 induced a different, more subtle response. We analyzed this gene expression dataset using our novel conditional clustering algorithm with the objective to identify genes that have a unique response to specific stimulations. The initial clustering found a total of 22 clusters [see Additional file 1] and the optimal polynomial order was 4. In our expression profile analysis, we observed multiple clusters, with a notable convergence of profiles for clusters 6, 8 and 12–14, in that all these clusters group the response induced by three distinct stimulation conditions (i.e., CD3/CD28, PHA, PMA/ionomycin), see Figure 9.

**Figure 10:**
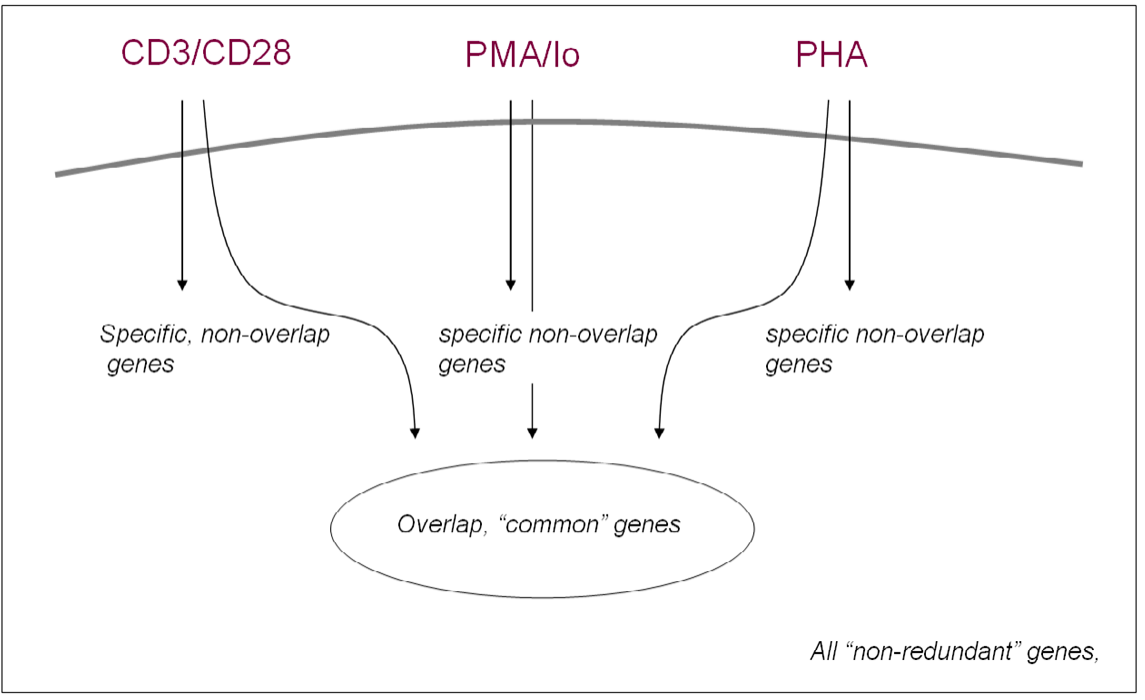
Schematic.

Given the similarity in profiles, we were interested in determining whether our analytical approach would be useful in systematically characterizing biological categories present within the unique and commonly expressed gene sets responsive to these stimuli (see schematic in Figure 10). Therefore, we applied the iterative procedure on these 22 clusters and found 13 clusters of GUP and 16 clusters of GCP. Among these 13 clusters, we see that there are four clusters solely from PMA/lo condition. Figure A.1 [See additional file 2] displays these four clusters, which are enriched for various biological functions including immune response and cell proliferation.

**Figure 11:**
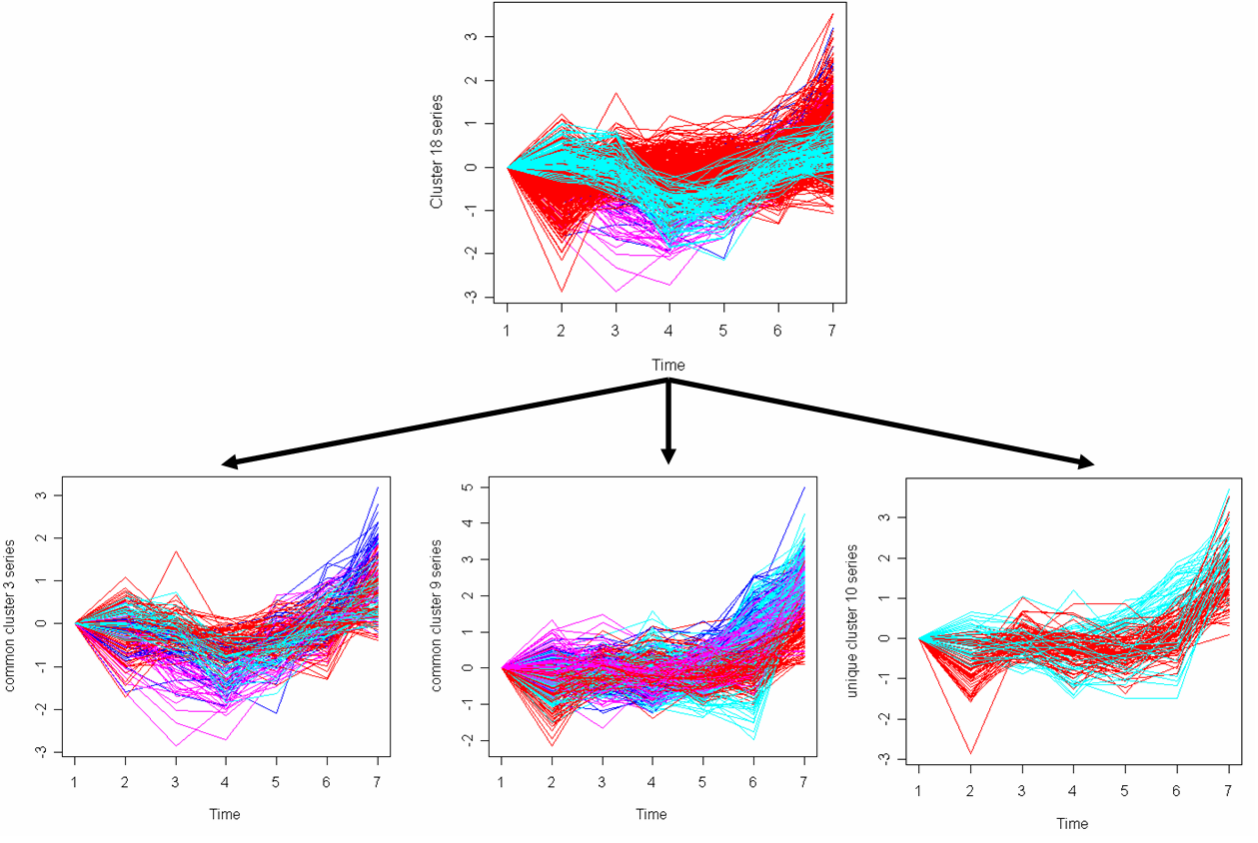
**Discovery of more subtle patterns with iterative procedure.** Red: expression of genes after stimulation of CD3; Pink: expression of genes after stimulation of PMA/lo; Blue: expression of genes after stimulation of PHA.

Most profiles from the initial 22 clusters are assigned to clusters of GCP and GUP in bulk [see Additional file 1]. For example, initial-cluster-7 has 118 profiles in GCP-cluster-15 and 22 profiles in GUP-cluster-9. There is one profile that is allocated to GUP-cluster-2, but it is ignorable. One interesting observation from this correspondence analysis is that some clusters in the initial analysis might be merging too many profiles, and by the iterative procedure, we can discover more subtle patterns. For example, a large amount of expression profiles of initial-cluster-18 are allocated to GCP-cluster-3, GCP-cluster-9 and GUP-cluster-10. Figure 11 shows these clusters. From this figure we see that initial-cluster-18, which is the cluster on the top, has a pattern that changes expression at the fourth time point and a pattern that changes expression at the sixth time point. With the iterative procedure, most of the profiles that change expression at the fourth time point are placed in GCP-cluster-3 (left in second row), and most of the profiles that change expression at the sixth time point are put into GCP-cluster-9 (middle in second row) and GUP cluster-10 (right in second row).

We conducted gene set enrichment analysis on the overall composition of genes by generating a non-redundant list of genes present within the five largest clusters of the initial analysis; an additional list of those genes that were shared or common to each of the distinct simulation conditions; and an additional list of those genes that were specifically stimulated by each condition: CD3/CD28 stimulation, PMA/ionomycin, and PHA, but that were not common to all three stimulations. The gene set enrichment analysis was conducted on each gene set using the software package EASE [29]. Notably, in the overall composition, we observed significantly enriched biological categories (both Gene Ontology categories and PubMed – based categories) that were predominantly related to cell division (DNA synthesis, cell cycle) and regulation of immune target gene expression (e.g., TFs and Immune). In the common gene set, we observed categories that were predominantly restricted to cell division (i.e., no categories for TF and immune, see Tables 2 and 3), suggesting that what these three stimuli have in common is their capacity to promote cell cycling and DNA synthesis. Finally, we observed enrichment for cell division and immune target gene expression specifically for CD3/CD28; but not with the other two stimuli, consistent with the larger analysis that included all non-redundant genes within the five clusters and with the trend observations made by Diehn and colleagues [21] that emphasized the predominant role for CD3/CD28 signaling. Of particular note, we observed a striking statistical enrichment of key immune transcription factor genes (e.g., NFKB2, NFATC1 and RELB) in the CD3/CD28 analysis. This observation was in contrast with the other two stimulations, and with the common, "overlapping" gene set (see Table 3). Overall, these results suggest that the methodology utilized in this study is useful in identifying and distinguishing uniquely stimulated genes from commonly stimulated genes in response to variable stimuli. The capacity to define specificity of gene expression response profiles is likely to be very useful in various biomedical efforts that are focused on more precisely defining both specific and common expression patterns in response to distinct stimuli.

**Table 2:** Biological categories identified by EASE. NS = not significant

Category analysis with EASE	Total genes in clusters	Common genes in clusters	Specific non-overlapping genes for each stimulation		
	All	Overlap	CD3/CD28	PMA/lo	PHA
	
DNA binding	0.03	NS	0.07	NS	0.009
TF and Immune-Hs	0.03	NS	0.007	NS	0.06
TF-immune-DNA binding-Hs	NS	NS	0.007	NS	NS
TF binding	0.03	NS	0.048	NS	NS
DNA synthesis	e-5	0.0004	0.003	0.003	0.03
Chromatin	0.01	NS	0.02	0.047	0.001
Cell cycle	e-23	e-7	e-13	e-13	e-17

**Table 3:** Gene Ontology categories

ENRICHED CATEGORIES	GO CATEGORY: TFS AND IMMUNE
Categories of overlapping genes in ALL stimulations: DNA synthesis, cell cycle	None
Categories of genes in ALL stimulations: DNA binding, TFs and immune, TF binding, DNA synthesis, cell cycle	CEBPG; CHUK; CXCR4; HIF1A; HLA-E; ICAM1; IL8; IRAK1; IRF2; NFATC1; NFKB2; POU2F2; RELB; STAT1; STAT5B; TBX21; TCF7; VDR; VIPR1; WT1
Categories of CD3/CD28 specific genes (overlapping genes were subtracted): TF and immune, DNA binding, TF binding, chromatin, DNA synthesis, DNA binding, RNA processing, cell cycle	CEBPG; CHUK; HIF1A; ICAM1; IL8; IRAK1; NFATC1; NFKB2; POU2F2; RELB; STAT1; STAT5B; TBX21; VDR; WT1

## Conclusion

Temporal microarray experiments across several experimental conditions are frequently conducted, and it is very important to recognize the specific features built in this design. In this paper we introduce a clustering technique that incorporates the experimental conditions in the analysis. More importantly, we develop an iterative procedure to distinguish, from a set of resulting clusters, clusters of common genes and unique genes. Our simulation study shows that this clustering algorithm can correctly identify the number of clusters, and find the common and unique genes with low error rate. The application of this technique to the analysis of data from [21] gives us a set of very interesting clusters biologically, and a new perspective to look at this data.

There are some limitations in this work that can stimulate further research. Other methods, for example bi-clustering, can be applied to cluster data measured in two conditions. Bi-clustering has the advantage of simultaneously clustering rows and columns, and it has been applied to co-clustering of genes and experimental conditions to find differentially expressed genes [30]. However, the current versions of bi-clustering do not take into account the temporal nature of the data. Nevertheless, it would be interesting to compare the approaches. Another limitation of this work is that we considered the situation in which the sampling frequency is the same across experimental conditions. It is not straightforward to generalize our approach to experiments comparing expression profiles measured with different sampling frequency. One possibility is to make the sampling frequencies homogeneous by adding the time points that are missing and use, for example, imputations to fill in the missing observations or Markov Chain Monte Carlo methods to estimate the Bayesian score.

## Availability and requirements

Project name: Bayesian conditional clustering of temporal expression profiles.

Project home page: .

Operating system: Microsoft Windows XP and Vista.

Programming language: R

License: GNU

## Authors' contributions

PS designed the study and oversaw the method development. LW developed, implemented and tested the method. MM suggested the idea of looking for GCP and GUP and MM and MR helped with the interpretation of the results. PS and LW drafted the manuscript. All the authors have revised and approved the final version of the manuscript.

## Supplementary Material

Additional file 1 **Table A.1**. The relation between the initial 22 clusters found in the data from Diehn et al. (column 1) and the clusters of GUP and GCP obtained by applying the iterative clustering procedure (column 2). The numbers in the parenthesis are the profiles assigned to the clusters of GUP or GCP. Click here for file

Additional file 2 **Figure A.1**. Clusters of GUPs for condition PMA/lo. We used different ranges of values in the y-axis to better describe the cluster profiles. Below each individual cluster plot are the enriched GO categories and the EASE score. Click here for file
