# Supplementary material for: Conditional clustering of temporal expression profiles
Source: BMC Bioinformatics. 2008 Mar 11;9:147. doi: 10.1186/1471-2105-9-147 (PMC2335301; doi:10.1186/1471-2105-9-147)
Supplement: Additional file 1 — Table A.1. The relation between the initial 22 clusters found in the data from Diehn et al. (column 1) and the clusters of GUP and GCP obtained by applying the iterative clustering procedure (column 2). The numbers in the parenthesis are the profiles assigned to the clusters of GUP or GCP. [file 1471-2105-9-147-S1.pdf]

Initial-cluster	Clusters after Iterative Procedure
Initial-cluster-1	GCP-cluster-9 (31), GUP-cluster-10 (6)
Initial-cluster-2	GCP-cluster-15 (3), GUP-cluster-2 (42)
Initial-cluster-3	GCP-cluster-16 (4), GUP-cluster-3 (2), GUP-cluster-4 (15)
Initial-cluster-4	GCP-cluster-7 (9), GCP-cluster-8 (246), GUP-cluster-6 (29), GUP-cluster-7 (129)
Initial-cluster-5	GCP-cluster-4 (2042), GCP-cluster-6 (108), GCP-cluster-7 (385), GUP-cluster-5 (256) GUP-cluster-6 (9), GUP-cluster-8 (20)
Initial-cluster-6	GCP-cluster-3 (3), GCP-cluster-7 (11), GCP-cluster-8 (29), GCP-cluster-9 (331) GCP-cluster-12 (1), GUP-cluster-7 (12), GUP-cluster-10 (40)
Initial-cluster-7	GCP-cluster-15 (118), GUP-cluster-2 (1), GUP-cluster-9 (22)
Initial-cluster-8	GCP-cluster-7 (3) , GCP-cluster-8 (740), GCP-cluster-9 (30), GCP-cluster-12 (6) GUP-cluster-6 (2), GUP-cluster-7 (167), GUP-cluster-10 (2)
Initial-cluster-9	GCP-cluster-10 (114), GCP-cluster-15 (8), GUP-cluster-1 (77), GUP-cluster-9 (31)
Initial-cluster-10	GCP-cluster-1 (563), GCP-cluster-4 (6), GCP-cluster-6 (26), GCP-cluster-7 (7) GCP-cluster-8 (50), GCP-cluster-9 (3), GCP-cluster-11 (2), GCP-cluster-13 (7) GUP-cluster-6 (31), GUP-cluster-7 (28), GUP-cluster-8 (10), GUP-cluster-12 (21) GUP-cluster-13 (2)
Initial-cluster-11	GCP-cluster-3 (1), GCP-cluster-4 (75), GCP-cluster-6 (830), GCP-cluster-7 (2) GCP-cluster-10 (4), GCP-cluster-14 (3), GCP-cluster-15 (6), GUP-cluster-5 (10) GUP-cluster-6 (2), GUP-cluster-8 (328), GUP-cluster-9 (2)
Initial-cluster-12	GCP-cluster-2 (92), GCP-cluster-5 (1), GCP-cluster-8 (21), GCP-cluster-9 (10) GCP-cluster-11 (1), GCP-cluster-12 (27), GUP-cluster-7 (22), GUP-cluster-11 (5)
Initial-cluster-13	GCP-cluster-8 (1), GCP-cluster-11 (192), GCP-cluster-12 (3), GCP-cluster-13 (13) GUP-cluster-13 (58)
Initial-cluster-14	GCP-cluster-6 (1), GCP-cluster-10 (24), GCP-cluster-15 (354), GUP-cluster-9 (145)
Initial-cluster-15	GCP-cluster-6 (1), GCP-cluster-7 (1), GCP-cluster-8 (85), GCP-cluster-9 (1) GCP-cluster-11 (13), GCP-cluster-12 (1154), GCP-cluster-13 (18), GUP-cluster-6 (1) GUP-cluster-7 (139), GUP-cluster-12 (38), GUP-cluster-13 (6)
Initial-cluster-16	GCP-cluster-5 (13), GCP-cluster-9 (5), GUP-cluster-7 (1), GUP-cluster-11 (2)
Initial-cluster-17	GCP-cluster-4 (40), GCP-cluster-6 (2), GCP-cluster-7 (1482), GCP-cluster-8 (68) GUP-cluster-5 (42), GUP-cluster-6 (188), GUP-cluster-7 (2)
Initial-cluster-18	GCP-cluster-3 (191), GCP-cluster-4 (4), GCP-cluster-6 (66), GCP-cluster-7 (25) GCP-cluster-8 (18), GCP-cluster-9 (82), GCP-cluster-10 (1), GUP-cluster-1 (2) GUP-cluster-6 (84), GUP-cluster-7 (7), GUP-cluster-8 (52), GUP-cluster-10 (77)
Initial-cluster-19	GCP-cluster-14 (36), GCP-cluster-15 (16), GUP-cluster-2 (1), GUP-cluster-9 (9)
Initial-cluster-20	GCP-cluster-11 (5), GCP-cluster-16 (40), GUP-cluster-13 (4)
Initial-cluster-21	GCP-cluster-6 (36), GCP-cluster-7 (1), GCP-cluster-10 (1564), GCP-cluster-14 (3) GCP-cluster-15 (71), GUP-cluster-1 (8), GUP-cluster-8 (19), GUP-cluster-9 (47)
Initial-cluster-22	GCP-cluster-7 (1), GCP-cluster-11 (6), GCP-cluster-13 (206), GUP-cluster-3 (29) GUP-cluster-4 (1), GUP-cluster-7 (1), GUP-cluster-12 (74), GUP-cluster-13 (15)

Table A.1
