# Supplementary material for: Conditional clustering of temporal expression profiles
Source: BMC Bioinformatics. 2008 Mar 11;9:147. doi: 10.1186/1471-2105-9-147 (PMC2335301; doi:10.1186/1471-2105-9-147)
Supplement: Additional file 2 — Figure A.1. Clusters of GUPs for condition PMA/lo. We used different ranges of values in the y-axis to better describe the cluster profiles. Below each individual cluster plot are the enriched GO categories and the EASE score. [file 1471-2105-9-147-S2.pdf]

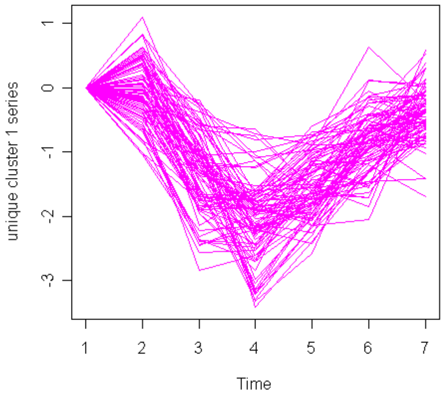


GUP-cluster-1
Catalytic activity
$(9.92 \times e^{-3})$

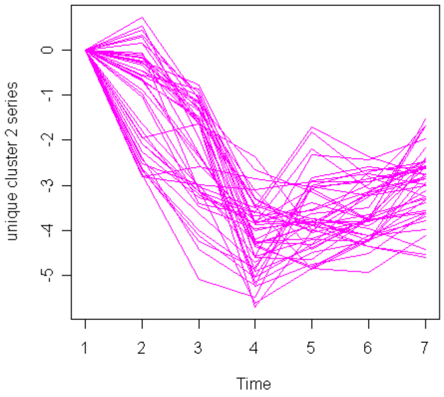


GUP-cluster-2
Plasma membrane
$(1.05 \times e^{-2})$

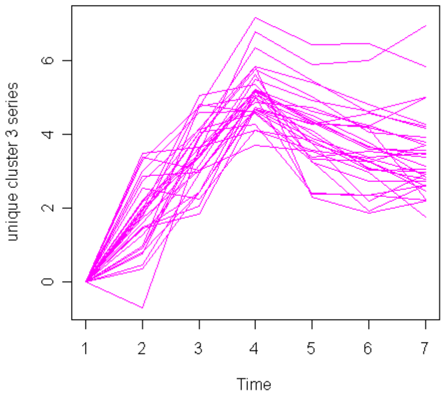


GUP-cluster-3
Positive regulation of cell proliferation
$(6.87 \times e^{-2})$

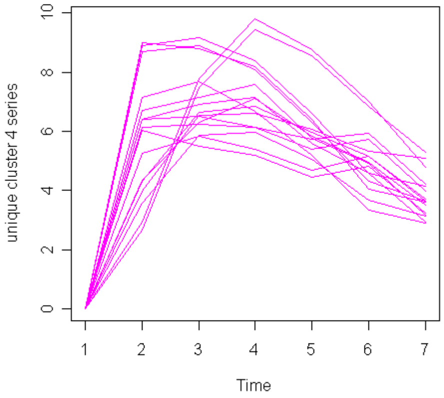


GUP-cluster-4
Immune response
$(8.48 \times e^{-3})$

Figure A.1
